# Analysis of the Associations between Vitamin D and Albuminuria or **β**-Cell Function in Chinese Type 2 Diabetes

**DOI:** 10.1155/2014/640909

**Published:** 2014-04-23

**Authors:** Xiaoling Cai, Zhaoheng Hu, Ling Chen, Xueyao Han, Linong Ji

**Affiliations:** Endocrine & Metabolism Department, Peking University People's Hospital, Beijing 100044, China

## Abstract

*Objective*. To investigate the associations of 25-(OH)D and ***β***-cell function or insulin resistance or albuminuria in Chinese type 2 diabetic patients. *Methods*. In total, 1408 type 2 diabetic patients without vitamin D supplement were included in this retrospective study. *Results*. Comparison between patients with and without 25-(OH)D deficiency indicated that, compared with patients with 25-(OH)D ≥ 50 nmol/L, patients with 25-(OH)D < 50 nmol/L showed a higher level of urine albumin-creatinine ratio (ACR) (90.15 ± 10.30 mg/g versus 52.79 ± 14.97 mg/g). Multiple regression analysis indicated that 25-(OH)D was independently and negatively correlated with urine ACR (OR = 0.985, 95%CI 0.972–0.999, *P* = 0.03), adjusted by age, diabetic duration, HBP duration, SBP, HbA1c, creatinine, LDL-C, triglyceride, total cholesterol, and HDL-C. Compared with patients with normal level of urine ACR, patients with higher level of urine ACR showed a significant lower level of 25-(OH)D (34.49 ± 13.52 nmol/L versus 37.46 ± 13.6 nmol/L, *P* = 0.00). Analysis of the associations of 25-(OH)D and ***β***-cell function or insulin resistance showed that 25-(OH)D may not correlate with ***β***-cell function or insulin resistance. *Conclusion*. 25-(OH)D was independently associated with albuminuria in Chinese type 2 diabetic patients but was not associated with ***β***-cell function or insulin resistance.

## 1. Introduction

Recently, accumulating evidence from several cross-sectional studies indicated that a factor that may impact the differential development of diabetic nephropathy is vitamin D. The role of the kidney in the hydroxylation of the vitamin D metabolite 25-OH-D from the liver to the biologically active form of 1,25-(OH)2D3 is well established [1, 2]. Animal studies suggested that vitamin D deficiency and insufficiency may have an active role in the progression of kidney disease [3–6]. Another interesting thing is that racial and ethnic differences exist in the prevalence of diabetic nephropathy [7, 8]. However, little information is available about the association between diabetic nephropathy and vitamin D levels in humans, especially in Chinese population.

Moreover, evidence from several cross-sectional studies also indicated that high serum 25-hydroxyvitamin D [25-(OH)D] concentration may be associated with lower risk of insulin resistance, high level of insulin secretion, and lower risk of type 2 diabetes [9–14]. However, reports on associations between insulin secretion or insulin resistance and 25-(OH)D have been inconsistent. What's more, how about the relation between insulin secretion or insulin resistance and 25(OH)D level in established diabetes patients? Few studies have examined these relationships in Asians especially in the Chinese.

Therefore, in the current study, we make two hypotheses: one is that there might be an association between vitamin D and albuminuria in Chinese type 2 diabetes patients; the other is that vitamin D might be inversely associated with insulin resistance and**β**-cell dysfunction in established Chinese type 2 diabetes population.

## 2. Materials and Methods

### 2.1. Patients

By searching inpatient database at Peking University People's Hospital, we have identified 1408 type 2 diabetic patients who were hospitalized for treatment at the ward of Department of Endocrinology and Metabolism of Peking University People's Hospital from January 2009 to December 2012. From that period of time, patients being hospitalized into our endocrinology department were all included in this retrospective study. Reasons for hospitalization were as follows: (1) advancement of oral hypoglycemic agents treatment or insulin treatment for better glycemic control, (2) optimizing the antidiabetic treatment and the treatment of diabetes complications, and (3) routine screening for diabetes complications by the patients' will. Patients with diabetic ketoacidosis or ketonuria and who tested positive for antiglutamic acid decarboxylase and islet cell antibody were excluded. Patients with routine vitamin D supplement or other treatment for osteoporosis before admission were excluded. The consent forms were all signed by the patients.

### 2.2. Ethics Statement

This is a retrospective study and the data were analysed anonymously; therefore, there is no need for informed consent. The ethics committee of Peking University People's Hospital has approved this retrospective study. All the patients were patients admitted at the Department of Endocrine & Metabolism of Peking University People's Hospital. On the first day of their admission, the patients signed the consent form for allowing their information to be stored in the hospital database and used for research. This consent form was also approved by the ethics committee of Peking University People's Hospital.

### 2.3. Variable Assessment

Anthropometric measurements at admission were collected; biochemical measurements including HbA1c (Primus ultra2, Primus Diagnostics, MO, USA), urinary albumin-creatinine ratio (COBAS Integra 400 Plus System, Roche Diagnostics Ltd., Basel, Switzerland), immunoreactive fasting insulin (Elecsys 2010 system, Roche Diagnostics Ltd., Basel, Switzerland), levels of total cholesterol (CHO), low-density lipoprotein cholesterol (LDL-C), high-density lipoprotein cholesterol (HDL-C), and triglyceride (COBAS Integra 400 Plus System, Roche Diagnostics Ltd., Basel, Switzerland) were assessed. Serum 25-(OH)D was determined by competitive RIA using the IDS RIA kit (Medicorp Inc.).

The homeostasis model assessment of insulin resistance (HOMA-IR) and HOMA-B were derived from fasting glucose and insulin levels. HOMA-IR was calculated using the formula fasting glucose (mmol/L) × fasting insulin (pmol/L)/22.5; HOMA-B was calculated using 20 × fasting insulin (pmol/L)/(fasting glucose (mmol/L) −3.5).

### 2.4. Definition of Albuminuria and Vitamin D Deficiency

Urinary albumin-to-creatinine ratio (ACR) was tested in three consecutive days for each patient, and the average ACR was calculated. The urine samples were collected in the early morning each day. Albuminuria was defined as urinary albumin-to-creatinine ratio ≥30 mg/g. Serum 25-hydroxycalciferol vitamin D levels were characterized as <50 nmol/L vitamin D deficiency, 50 to 75 nmol/L vitamin D insufficiency, and >75 nmol/L normal vitamin D.

### 2.5. Statistical Analysis

Variables were represented as means ± s.d. Continuous variables were compared using a two-sample* t* test while frequency of dichotomous variables was performed by *χ* ^2^ analysis. A two-sided *P* ≤ 0.05 was considered significant. Multivariable logistic regression analyses were made to assess the correlation between serum 25-(OH)D (VitD) and HOMA-IR, HOMA-B, or urine ACR. All *P* values were 2-tailed and considered significant at *P* < 0.05. Regression analyses were performed using SPSS 19.0.

## 3. Results

### 3.1. Clinical and Metabolic Parameters of Patients

Clinical characteristics of patients were shown in Table 1. The average age was 57.09 ± 13.54 years old; the average diabetic duration was 9.39 ± 7.73 years. The average HbA1c was 8.95 ± 2.11%. The level of 25-(OH)D was 35.72 ± 13.64 nmol/L. The average HOMA-IR was 5.28 ± 7.49 and HOMA-B was 119.37 ± 409.89. The average urine ACR was 84.21 ± 293.12 mg/g.

### 3.2. Comparisons between Patients with Different Level of 25-(OH)D

Patients with 25-(OH)D deficiency occupied 84.4%, while patients with 25-(OH)D insufficiency occupied 14.16%, only 1.44% patients with normal level of 25-(OH)D. Comparisons between patients with and without 25-(OH)D deficiency indicated that, compared with patients with 25-(OH)D ≥ 50 nmol/L, patients with 25-(OH)D < 50 nmol/L showed a higher level of HbA1c (9.03 ± 0.07% versus 8.55 ± 0.16%, *P* = 0.01), a higher level of TG (1.96 ± 0.05 mmol/L versus 1.65 ± 0.08 mmol/L, *P* = 0.02), and a higher level of urine ACR (90.15 ± 10.30 mg/g versus 52.79 ± 14.97 mg/g) (Table 1).

### 3.3. Association between Serum 25-(OH)D Concentration and Albuminuria

Then, multiple logistic regression analysis was used to identify the association between serum 25-(OH)D concentration and urine ACR. Urine ACR was used as dependent variable, gender, diabetic duration, age, and season of the examination as independent variables in model 1, model 1 and BMI, SBP, and HbA1c as independent variables in model 2, and model 2 and LDL-C, HDL-C, triglyceride, total cholesterol, CRE, and UA level as independent variables in model 3. Results showed that serum 25-(OH)D concentration was associated with urine ACR in each model (model 1: OR = 0.984, 95% CI 0.972–0.996, *P* = 0.008; model 2: OR = 0.985, 95% CI 0.973–0.998, *P* = 0.021; model 3: OR = 0.985, 95% CI 0.972–0.999, *P* = 0.030). The lower level of serum 25-(OH)D was associated with the high level of urine ACR. Details were shown in Table 2. Compared with patients with normal level of urine ACR, patients with higher level of urine ACR showed a significant lower level of 25-(OH)D (34.49 ± 13.52 nmol/L versus 37.46 ± 13.6 nmol/L, *P* = 0.00). Details were shown in Table 2.

### 3.4. Association between Serum 25-(OH)D Concentration and Insulin Resistance

Then, multiple logistic regression analysis was used to identify the association between serum 25-(OH)D concentration and insulin resistance. HOMA-IR was used as dependent variable, gender, diabetic duration, age, and season of the examination as independent variables in model 1, model 1 and BMI, SBP, and HbA1c as independent variables in model 2, and model 2 and LDL-C, HDL-C, triglyceride, total cholesterol, CRE, UA level, and urine ACR as independent variables in model 3. Results showed that serum 25-(OH)D concentration was not associated with HOMA-IR in each model. Details were shown in Table 2.

### 3.5. Association between Serum 25-(OH)D Concentration and**β**-Cell Function

Then, multiple logistic regression analysis was used to identify the association between serum 25-(OH)D concentration and**β**-cell function. HOMA-B was used as dependent variable, gender, diabetic duration, age, and season of the examination as independent variables in model 1, model 1 and BMI, SBP, and HbA1c as independent variables in model 2, and model 2 and LDL-C, HDL-C, triglyceride, total cholesterol, CRE, UA level, and urine ACR as independent variables in model 3. Results showed that serum 25-(OH)D concentration was not associated with HOMA-B in each model. Details were shown in Table 2.

## 4. Discussion

This study suggested independent association of 25-(OH)D with albuminuria but did not find the associations of insulin sensitivity and**β**-cell function with 25-(OH)D in Chinese type 2 diabetes patients.

The major contribution of this study is the finding of an association between vitamin D and urine ACR, an index of diabetic nephropathy in Chinese type 2 diabetes patients. Multiple logistic regression analysis indicated that serum 25-(OH)D concentration was inversely correlated with urine ACR. In Caucasian patients, Diaz et al. [15] reported according to a cross-sectional analysis of the 2001 to 2006 National Health and Nutrition Examination Survey that higher proportions of individuals with nephropathy have vitamin D deficiency than individuals without nephropathy (53.2% versus 47.0%, *P* < 0.03). They also demonstrated that vitamin D deficiency and insufficiency were associated with the presence of nephropathy (odds ratio, 1.85; 95% CI, 1.06–3.23 for vitamin D deficiency; and odds ratio, 1.79; 95% CI, 1.12–2.85 for vitamin D insufficiency), the results of which were in accordance with ours. However, because of the nature of this retrospective study, we were unable to determine whether this association is present because vitamin D deficiency increases the risk of nephropathy or because nephropathy increases the risk of vitamin D deficiency. This study evaluates 25-OH-D, as the NHANES study, which is the circulating metabolite produced in the liver that is later metabolized in the kidneys to 1,25-(OH)2D3. Based on this well-established pathway, renal insufficiency could not be the reason for the low levels of 25-OH-D seen in the study. This suggests that studies to further describe the role of vitamin D as a possible risk marker or risk factor for albuminuria and diabetic nephropathy are needed to evaluate the impact of maintaining an adequate level of vitamin D on the progression of diabetic nephropathy.

In terms of the association of vitamin D and beta cell function and insulin resistance, there are several potential effects of vitamin D on pancreatic beta cell function and insulin action [16–21]. The direct effect of vitamin D may be mediated by binding of its circulating active form 1,25-(OH)2D3 to the beta cell vitamin D receptor. The indirect effects of vitamin D may be mediated via its role in regulating extracellular calcium and calcium flux through the beta cell. Changes in calcium influx in primary insulin target tissues may contribute to peripheral insulin resistance. However, reports on associations between insulin secretion or insulin resistance and vitamin D have been inconsistent [22–30]. According to this study in Chinese type 2 diabetes patients, the level of 25-(OH)D may not be associated with HOMA-B, an index of pancreatic beta cell function derived from fasting insulin and glucose concentrations, and may not be associated with HOMA-IR, an index of insulin resistance. Boucher and others in several cohorts [22–26] reported that hypovitaminosis D is associated with beta cell dysfunction, but not in Lind et al. [27] and Orwoll et al. [28] studies. Baynes and others reported in several cross-sectional studies that there were associations between low vitamin D level and decreased insulin sensitivity [23–30], but not in Orwoll et al. [28] study. The differences in this relationship are likely due to differences in subject populations and disparate methods to determine insulin secretion.

In general, this retrospective study has shown some limitations. This study investigating relationships between 25-(OH)D and insulin secretion and sensitivity used indirect proxy measures, as HOMA-IR and HOMA-B. The accuracy of proxy measures of insulin sensitivity may vary depending on obesity status. In addition, although more than a thousand patients were included in this study, the sample size for evaluating the association between 25-(OH)D and insulin secretion and sensitivity may be small. However, as far as we know, this study may be the first study in Chinese type 2 diabetes patients to assess the relationship of 25-(OH)D and insulin secretion and sensitivity as well as the relationship of 25-(OH)D and urine ACR. Furthermore, the nature of this retrospective study results in the limitation to infer causation.

In conclusion, this study suggested that 25-(OH)D was significantly correlated with albuminuria in Chinese type 2 diabetic patients but may not be associated with**β**-cell function or insulin resistance.

## Figures and Tables

**Table 1:** Comparisons between patients with different level of 25-(OH)D.

	All patients	25-(OH)D < 50 nmol/L	25-(OH)D ≥ 50 nmol/L	*P* value
Number of patients	1408	1097	311	
Gender (male/female)	57.4%/42.6%	59.6%/40.4%	65.9%/34.1%	0.09
Age (years)	57.1 ± 13.54	57.5 ± 12.9	57.0 ± 12.5	0.63
Duration of DM (years)	9.4 ± 7.7	9.4 ± 7.8	10.1 ± 7.4	0.29
BMI (kg/m^2^)	25.24 ± 4.00	25.38 ± 3.80	25.27 ± 3.44	0.72
Waist (cm)	91.9 ± 12.1	92.1 ± 11.6	90.9 ± 13.6	0.24
SBP (mmHg)	131.7 ± 17.3	131.1 ± 17.4	132.3 ± 15.2	0.59
DBP (mmHg)	78.8 ± 11.2	78.7 ± 11.4	80.2 ± 10.48	0.13
25-(OH)D (nmol/L)	35.72 ± 13.64	32.41 ± 8.02	60.22 ± 11.20	0.00
HbA1c (%)	8.95 ± 2.11	9.03 ± 2.10	8.55 ± 2.05	0.01
Fasting glucose (mmol/L)	8.07 ± 2.83	8.17 ± 2.83	7.70 ± 2.80	0.01
Fasting insulin (uU/mL)	14.65 ± 18.95	14.98 ± 19.17	12.90 ± 17.66	0.27
HOMA-B	119.37 ± 409.89	124.68 ± 454.9	91.91 ± 168.3	0.41
HOMA-IR	5.28 ± 7.49	5.41 ± 7.90	4.63 ± 5.75	0.30
Postprandial insulin (uU/mL)	62.59 ± 119.61	62.24 ± 112.7	64.46 ± 152.1	0.85
UA (umol/L)	313.5 ± 90.3	312.2 ± 89.6	329.8 ± 92.0	0.02
CRE (umol/L)	63.48 ± 31.26	62.09 ± 37.43	61.34 ± 18.18	0.79
CHO (mmol/L)	4.74 ± 1.19	4.76 ± 1.19	4.59 ± 1.11	0.12
TG (mmol/L)	1.88 ± 1.58	1.96 ± 1.70	1.65 ± 1.01	0.02
HDL-C (mmol/L)	1.17 ± 2.82	1.06 ± 0.41	1.08 ± 0.32	0.42
LDL-C (mmol/L)	2.65 ± 0.84	2.70 ± 0.82	2.68 ± 0.88	0.75
hsCRP (mg/L)	3.0 ± 3.4	3.1 ± 3.0	2.5 ± 3.4	0.09
Urine ALB (mg/g)	84.2 ± 293.1	90.1 ± 305.3	52.8 ± 243.0	0.04

**Table 2:** Associations between serum 25-(OH)D concentration and insulin resistance, **β**-cell function, and urine ACR in Chinese type 2 diabetes.

	OR	95% CI	*P* value
25-(OH)D concentration (nmol/L) and HOMA-IR*			
Model 1	1.012	1.002–1.024	0.025
Model 2	1.009	0.996–1.021	0.169
Model 3	1.009	0.997–1.022	0.150

25-(OH)D concentration (nmol/L) and HOMA-B**			
Model 1	0.999	0.988–1.010	0.830
Model 2	0.994	0.983–1.006	0.336
Model 3	0.992	0.981–1.004	0.206

25-(OH)D concentration (nmol/L) and urine ACR (mg/g)***			
Model 1	0.984	0.972–0.996	0.008
Model 2	0.985	0.973–0.998	0.021
Model 3	0.985	0.972–0.999	0.030

*Model 1: adjusted by gender, age (year), duration of diabetes (year), and season; model 2: adjusted by model 1 + BMI (kg/m^2^), SBP (mmHg), and HbA1c (%); model 3: adjusted by model 2 + urine ACR (mg/g), LDL-C (mmol/L), HDL-C (mmol/L), CHO (mmol/L), TG (mmol/L), UA (umol/L), and CRE (umol/L). HOMA-IR > 3.30 as 1, while HOMA-IR < 3.30 as 2.

**Model 1: adjusted by gender, age (year), duration of diabetes (year), and season; model 2: adjusted by model 1 + BMI (kg/m^2^), SBP (mmHg), and HbA1c (%); model 3: adjusted by model 2 + urine ACR (mg/g), LDL-C (mmol/L), HDL-C (mmol/L), CHO (mmol/L), TG (mmol/L), UA (umol/L), and CRE (umol/L). HOMA-B < 50 as 1, while HOMA-B > 50 as 2.

***Model 1: adjusted by gender, age (year), duration of diabetes (year), and season; model 2: adjusted by model 1 + BMI (kg/m^2^), SBP (mmHg), and HbA1c (%); model 3: adjusted by model 2 + LDL-C (mmol/L), HDL-C (mmol/L), CHO (mmol/L), TG (mmol/L), UA (umol/L), and CRE (umol/L). Urine ACR < 30 mg/g as 1, while urine ACR ≥ 30 mg/g as 2.

## Abbreviations

25-(OH)D: 25-Hydroxyvitamin D
95% CI: 95% confidence interval
ACR: Albumin-creatinine ratio
BMI: Body mass index
CHO: Total cholesterol
CRE: Creatinine
CRP: C reactive protein
DBP: Diastolic blood pressure
DM: Diabetes mellitus
HbA1c: Hemoglobin A1c
HBP: Hypertension
HDL-C: High-density lipoprotein cholesterol
HOMA-B: Homeostasis model assessment of**β**-cell function
HOMA-IR: Homeostasis model assessment of insulin resistance
LDL-C: Low-density lipoprotein cholesterol
OR: Odds ratio
SBP: Systolic blood pressure
TG: Triglyceride
UA: Uric acid
WHR: Waist hip ratio.
